# Optimising the sensing volume of OPM sensors for MEG source reconstruction

**DOI:** 10.1016/j.neuroimage.2022.119747

**Published:** 2022-11-18

**Authors:** Yulia Bezsudnova, Anna U. Kowalczyk, Lari M. Koponen, Giovanni Barontini, Ole Jensen

**Affiliations:** University of Birmingham, UK

## Abstract

Magnetoencephalography (MEG) based on optically pumped magnetometers (OPMs) has been hailed as the future of electrophysiological recordings from the human brain. In this work, we investigate how the dimensions of the sensing volume (the vapour cell) affect the performance of both a single OPM-MEG sensor and a multi-sensor OPM-MEG system. We consider a realistic noise model that accounts for background brain activity and residual noise. By using source reconstruction metrics such as localization accuracy and time-course reconstruction accuracy, we demonstrate that the best overall sensitivity and reconstruction accuracy are achieved with cells that are significantly longer and wider that those of the majority of current commercial OPM sensors. Our work provides useful tools to optimise the cell dimensions of OPM sensors in a wide range of environments.

## 1 Introduction

Magnetoencephalography (MEG) provides a powerful non-invasive tool for neuroimaging. Conventional MEG systems consist of arrays of hundreds of pick-up coils coupled to superconducting quantum interference devices (SQUIDs) and enable the reconstruction of neural activity with millisecond temporal precision [1] and millimeter resolution [2]. The requirement of cryogenics and thermal insulation, as well as lack of flexibility, are the main limiting factors for SQUIDs in biomagnetic measurements.

Optically pumped magnetometers (OPMs) [3] are an alternative type of sensitive magnetometers with the potential to surpass the performance of superconducting sensors in brain imaging. Successful demonstrations of detection of human brain activity using various types of OPMs have been reported over the past years [4, 5, 6, 7, 8, 9, 10, 11, 12]. A number of works demonstrated that OPMs enable the study of previously difficult to detect biomagnetic signals in human and animal neurophysiology [11, 13, 14, 15, 16, 17, 18].

In OPMs the thermal insulation gap is in the mm range and, as a consequence, the sensors can be brought closer to the scalp and the brain than SQUIDs. Based on simulations, the neuromagnetic signal measured by an on-scalp OPM sensor is expected to be on average 5 times stronger than the one measured by a SQUID sensor. This can potentially lead to more accurate source reconstruction [19, 20, 21]. Despite this potential, so far the experimental results have only demonstrated OPM-MEG performance comparable to conventional SQUID-MEG systems [22, 23, 24].

Recent works have studied various aspects of the performance of OPM-MEG systems, trying to unlock their full potential. Investigations focused on quantifying the dependence of neuronal source localisation accuracy on the number of sensors, their layouts [25, 26, 27, 28], improvement of information content acquired with multi-axial sensor arrays, [20, 29] or accuracy of sensor positioning needed for precise source reconstruction [22, 30]. Most of these works assumed fixed single-sensor response and noise models that are based on either idealized point-like sensors [19, 29, 31, 32] or sensors with arbitrary noise levels [20].

It is of capital importance to consider that neuromagnetic fields decay with the square of the distance and an OPM sensor registers the signal averaged over its sensitive volume. Therefore, a smaller sensing volume results in higher recorded signal amplitude but also higher recorded brain noise. Furthermore, the size of the sensitive volume affects the intrinsic sensitivity of the sensor. Existing OPMs have cell sizes ranging from 1 × 2 × 3 *mm*^3^ in chip-scale sensors [33] to bench-top sensors with 30 mm spherical cells [34]. So far, however, no exhaustive analysis has been carried out to determine the optimal sensing element dimensions for an OPM-MEG system.

In this work, we study how the dimensions of the OPM sensing element, namely its vapour cell, can be optimised depending on residual noise sources. We first concentrate on the optimisation of the performance of a stand-alone sensor, which can be useful for the development of prototype sensors. In this case, we determine the optimal dimensions by maximising the signal-to-noise ratio. We then consider arrays of sensors, determining their optimal sensing volume by optimising the ability of the array to localise a source and extract its time course.

## 2 Theory

This section is divided into two parts. The first part collects the equations that we use to study and optimise the dimensions of the vapour cell of a single OPM sensor; the second part describes the equations required to expand the analysis to an array of sensors.

### 2.1 Stand-alone OPM sensor

The core component of an OPM sensor is the vapour cell that contains the atomic gas, typically an alkali metal vapour. An OPM measures magnetic fields by measuring changes in the properties of light interacting with the atomic medium contained in its sensing volume, once this is exposed to such fields [35]. The performance of any magnetometer can be assessed by its signal-to-noise ratio,

(1) $$SNR=\frac{S}{\delta \tilde{B}},$$

where *S* is the signal amplitude detected by the sensor and $\delta \tilde{B}$ is the total amplitude of the recorded noise. In a perfectly shielded environment the value of $\delta \tilde{B}$ determines the sensitivity *δB* of the sensor in a given measurement bandwidth *f_BW_*, $\delta B=\delta \tilde{B}/\sqrt{f_{BW}}$^1^. In real conditions it is impossible to separate the external magnetic field fluctuations from the intrinsic noise of the sensor. Therefore, $\delta \tilde{B}$ can be expressed as:

(2) $$\delta \tilde{B}=\sqrt{{\left(\delta {\tilde{B}}_{i}\right)}^{2}+{\tilde{N}}^{2}},$$

with $\delta {\tilde{B}}_{i}$ the intrinsic noise of the sensor and $\tilde{N}$ the external magnetic field noise amplitude. There are two fundamental noise sources that determine the intrinsic sensitivity of an OPM:

(3) $$\delta B_{i}=\sqrt{{\left(\delta B_{at}\right)}^{2}+{\left(\delta B_{ph}\right)}^{2}},$$

where *δB_at_* is the atomic-shot noise and *δB_ph_* is the the photon-shot noise. The expressions for these two terms are derived in Appendix A for both nonlinear magneto-optical rotation (NMOR) and spin-exchange relaxation-free (SERF) OPM sensors. In this work, we assume *f_BW_* = 25 Hz as that is sufficient to record oscillatory brain activity in the alpha and beta band.

The external magnetic field noise amplitude recorded by the sensor in MEG experiments can be expressed as:

(4) $$\tilde{N}=\sqrt{{\left({\tilde{N}}_{b}\right)}^{2}+{\left({\tilde{N}}_{r}\right)}^{2}},$$

where ${\tilde{N}}_{b}$ is the detected “brain noise”, resulting from the background brain activity and ${\tilde{N}}_{r}$ is the residual magnetic field noise that accounts for the components of the environmental noise and sensor’s technical noise that cannot be compensated for. The brain noise decreases with the distance from the head, therefore ${\tilde{N}}_{b}$ depends on the volume of the cell. The environmental noise originates from stray magnetic fields, thermal currents induced in the magnetically shielded room and vibrations of its walls, nearby electrical equipment, mechanical movement of magnetic or conductive components (e.g. elevators, urban traffic), and electrically active tissues. Such noise, generated by distant sources outside of the head, can be considered as spatially homogeneous [17]. The spatially and temporally correlated components of the environmental noise can be usually removed in data pre-processing by using various filtering methods such as signal space separation (SSS) [15, 36] or with magnetic field compensation systems (both require additional reference sensors). Advanced coils, such as those described in [37], can attenuate the dominant components of the static background field as well as their first order spatial gradients. Additionally, if used with feedback controllers, very low frequency magnetic field drifts can be significantly suppressed. However, such compensation can introduce magnetic noise in other frequency bands. In addition, every OPM sensor is subject to technical noise arising from fluctuations in the laser light intensity, frequency and polarization fluctuations, atomic cell temperature fluctuations, current noise, and other various electronic noises that can affect the magnetometer readout [38]. In our simulations, ${\tilde{N}}_{r}$ accounts for every technical and residual noise that cannot be actively or passively compensated. We additionally consider ${\tilde{N}}_{r}$ as white noise in the spectral interval 4–100 Hz [39]. In line with state-of-the-art methods like those developed in [15, 40, 41], we assume that the noise below 4 Hz is filtered out, therefore our measurement bandwidth is 5-30 Hz. In this work, ${\tilde{N}}_{r}$ is used as a free parameter with the standard deviation ranging from 0 to 100 fT, similarly to [21].

#### 2.1.1 Forward model for an OPM sensor

Our signal of interest, S, and brain noise, ${\tilde{N}}_{b}$, are due to the magnetic field arising from the neural activity in the brain. This neural activity gives rise to a primary current distribution and we approximate it with a set of equivalent current dipoles (ECDs) inside the brain. We obtain the associated magnetic field from a spherical volume conductor model of the head, identical to the one in [1]. We approximate the signal *S* with one tangential source ECD and the brain noise with a set of independent, tangential and randomly oriented ECDs [20, 39, 42, 43].

**Figure 1:**
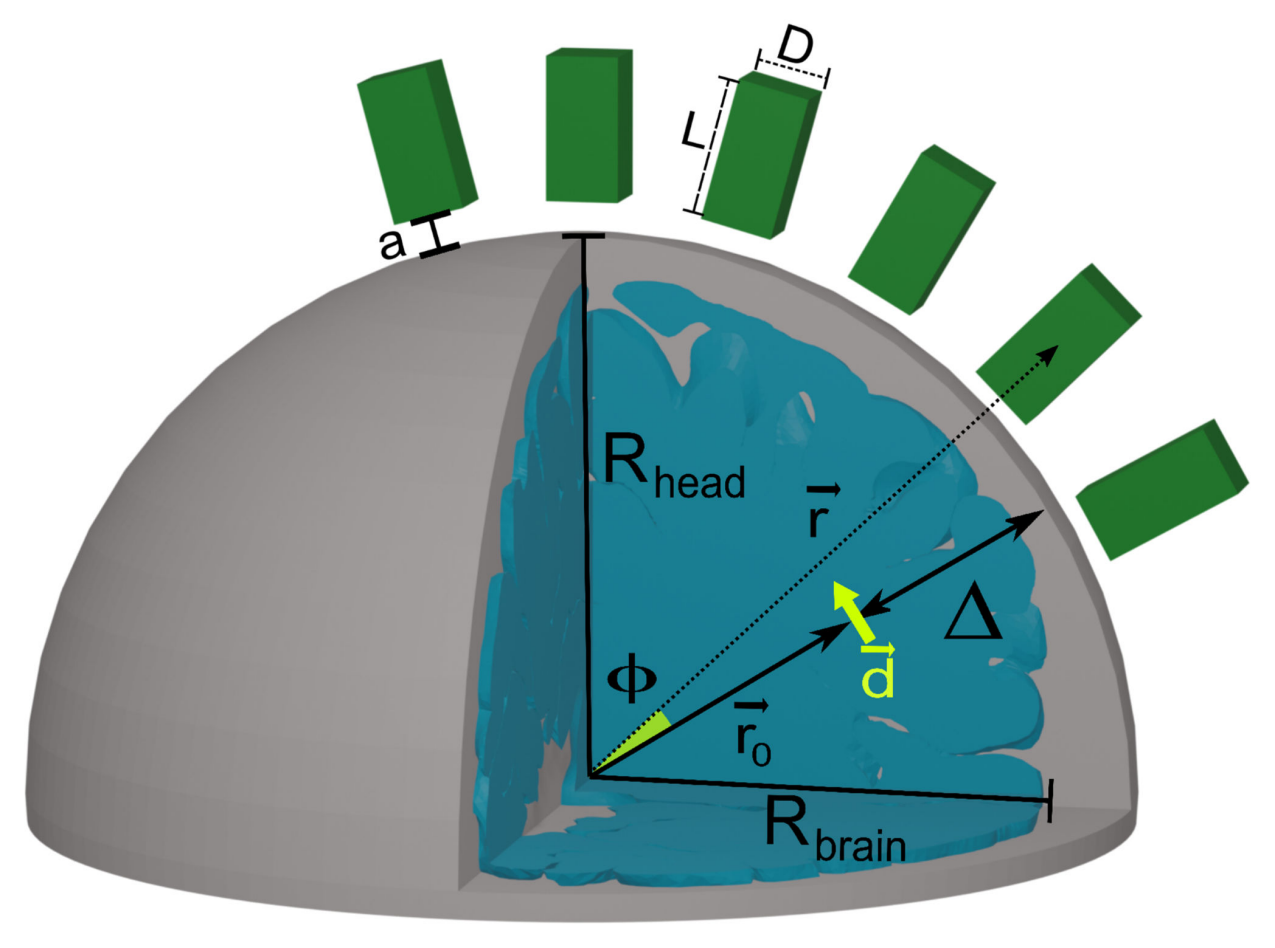
The model. The radius of the head is *R_head_* and the radius of the brain is *R_brain_*. An equivalent current dipole tangential to the head surface is positioned at point ${\vec{r}}_{0}$ inside the brain and has a moment $\vec{d}$. The distance between the head surface and the dipole is Δ. A sensor with a rectangular cuboid cell positioned at $\vec{r}$ is measuring a signal *S* from the dipole. The cuboid has length *L* in the radial direction and *D* in both tangential directions and is away from the head by distance *a*. The angle between ${\vec{r}}_{0}$ and $\vec{r}$ is *ϕ*.

As mentioned, an OPM produces a signal that is proportional to the mean magnetic field measured within the sensing volume [3]. For NMOR sensors, that typically use paraffin coated cells, this volume is determined by the glass cell volume, while for SERF sensors, that employ buffer gas cells, sensing volume is the intersection between the cell volume and the probe laser beam. Therefore the signal of interest is

(5) $$S=\frac{1}{V}\int_{V}{\vec{B}}_{sECD}(\vec{r})\cdot \vec{n}dV,$$

where *V* = *L* × *D*^2^ is the volume of the vapour cell with length *L* and the cross-section *D*^2^, ${\vec{B}}_{sECD}$ is the magnetic field produced by the source ECD, and $\vec{n}$ is the measurement axis of the OPM. Similarly, the brain noise is the net signal generated by a set of randomly oriented dipoles:

(6) $${\tilde{N}}_{b}=RMS_{100}\left[\underset{j}{\sum^{\text{​}}}\frac{1}{V}\int_{V}{\vec{B}}_{ECD,j}(\vec{r})\cdot \vec{n}dV\right],$$

where the index *j* runs over the dipoles. The noise amplitude is obtained by generating 100 of such sets and calculating the root mean square of the computed sums. The modeled system is shown in Fig. 1. The head is approximated with a conductive sphere with radius *R_head_* = 91 mm and the brain with a concentric sphere with radius *R_brain_* = 80 mm. The signal S arises from a single tangential 10 nAm dipole, and ${\tilde{N}}_{b}$ from 1000 randomly oriented dipoles (0.2 nAm each). For such model, the forward problem has a closed-form solution [44] and we obtain realistic values for the signal of interest and brain noise.

We define *L_opt_* and *D_opt_* as optimal length and width that yield the highest value of SNR at given *N_r_*. Our procedure to find *L_opt_* and *D_opt_* for a single OPM sensor is the following: (i) we generate a randomly oriented tangential source dipole at a given depth (Δ = 20–45 mm) and adjust the angle between the OPM sensing axis and the dipole *ϕ* (see Fig. 1) to maximise S. (ii) we generate 1000 randomly uniformly oriented and positioned ECDs inside the brain. (iii) we add a white residual noise *N_r_* and intrinsic noise $\delta {\tilde{B}}_{i}$. (iv) we search for the highest SNR scanning the sensor dimensions *L* and *D*. This procedure is repeated for 100 trials. For each set of dimensions we calculate $\delta {\tilde{B}}_{i}$ using the parameters listed in Appendix A. All computations are performed using the FieldTrip toolbox [45] and custom MATLAB scripts (R2019b, Mathworks, USA). We have verified that our model reproduces typical values. For an ideal noise-free point-like SQUID sensor placed 40 mm above the scalp, $N_{r}=5\;\text{fT}/\sqrt{Hz}$, and Δ =2.1 cm, we obtain SNR ≈ 1.9, which is within the range of typical MEG data [46].

### 2.2 An array of OPM sensors

We estimate the performance of an OPM-MEG system, consisting of an array of sensors, using its source localisation accuracy and time course reconstruction accuracy. A quasi-uniform array of sensors over one of the hemispheres of a spherically symmetric head model reaches best localisation accuracy with a finite number of sensors, depending on the amplitudes of both the brain noise ${\tilde{N}}_{b}$ and the signal of interest *S* [39]. In this work, we define the optimum number of sensors as the minimum number of sensors that enables best localisation accuracy. Assuming that each sensor in the array has identical sensing volume, we perform an exhaustive search over *L* and *D* to maximise the source reconstruction accuracy. For these calculations, ${\tilde{N}}_{r}$ is set to 25 fT, corresponding to $N_{r}=5\;\text{fT}/\sqrt{Hz}$. As long as the dimensions of the sensitive volume of each sensor are much smaller than the spacing between adjacent sensors, the spatial sampling of an array of sensors [47] is limited by the sensor spacing and not by dimensions of the sensitive volume. This greatly simplifies the problem because it is sufficient to optimise the sensitive volume to accurately reconstruct a single ECD, rather than modeling the general case of two or more partially correlated sources.

#### 2.2.1 Localisation accuracy

We define the localisation accuracy for a single dipolar source as the volumetric error

(7) $$\sigma_{V}=\sqrt{{\left(\sigma_{x}\right)}^{2}+{\left(\sigma_{y}\right)}^{2}+{\left(\sigma_{z}\right)}^{2}},$$

where *σ_i_* is the root mean square (RMS) error of the reconstructed dipole position in the direction *i* [39]. To avoid overfitting due to exactly ideal forward model, we introduce trial-by-trial inaccuracy to sensor positions and orientations. When measuring the field and the brain noise, each sensor has a uniform random offset (RMS 4 mm) and a uniform random tilt (RMS 9°) from the normal to the head orientation, corresponding to the suggested co-registration accuracy of an OPM-MEG system by [30]. Our procedure in this case is the following: (i) we generate a random tangential ECD in a random position (Δ = 21–26 mm) and 1000 randomly oriented ECDs inside the brain. (ii) we compute the signal and brain noise recorded by the inaccurately positioned sensor array (100 sets). (iii) we add white residual and intrinsic noise. (iv) assuming the ideal sensor locations, we reconstruct a single dipole using a dipole fitting algorithm ^2^. For each array of sensors, the localisation procedure was performed for 9 ECD dipole positions, repeated 100 times for different sensor location errors and noise $\tilde{N}$. We do maintain the correct spatial relationship between the sources and the generated brain noise.

#### 2.2.2 Time course reconstruction accuracy

The ability to localise a dipolar source does not reveal the full picture of the performance of an array of sensors, as such reconstruction has very low sensitivity to uncorrelated noise. Thus, to better assess the performance, we also compute the time course prediction error *E_tot_*, a metric that estimates the ability of the array to reconstruct the temporal waveform of a dipolar source using a beamformer analysis [48].

As our MEG model includes spatially correlated brain noise, we cannot use the closed-form solution of *E_tot_* derived by [29]. Instead we estimate *E_tot_* as

(8) $$E_{\text{tot}\;}=\frac{1}{M}\sqrt{\sum_{i=1}^{M}{\left({\hat{q}}_{i}-q_{i}\right)}^{2}},$$

where *M* = 10, 000 is the number of time points, ${\hat{q}}_{i}$ is the estimated source magnitude, and *q_i_* is the true source magnitude at time *i*. Here,

(9) $${\hat{q}}_{i}=\omega^{⊤}b_{i},$$

where ***ω*** is the filter to extract ${\hat{q}}_{i}$ from the measurement ***b_i_***. Given that

(10) $$\omega =\omega argmin\;E\;({\hat{q}}_{i}^{2})\;\text{while}\;\omega^{⊤}l=1\text{,}\;$$

where *E*(·) is the expected value and ***l*** is the lead field of the source. A closed-form solution is [49]:

(11) $$\omega =\frac{l^{⊤}{\text{C}}^{-1}}{l^{⊤}{\text{C}}^{-1}l},$$

where *C* is the data covariance matrix.We use the exact *C* as the sum of contributions from the source, all of the 1000 noise dipoles, *N_r_* and *δB_i_*. For each dipole $C^{*}={\left(q_{RMS}^{*}\right)}^{2}\cdot l^{*}l^{*⊤}$, where $q_{RMS}^{*}$ is the root mean square of the amplitude of the relevant dipole (marked with *) over time. While the contribution from all the other noise sources is given by C^*^ = *s* · *I*, where *s* is a standard deviation of the relevant noise term and *I* is the identity matrix. To reduce random variability, we show the root-mean-square value of 9 *E_tot_* estimates for random source dipoles located 21–26 mm from the head surface. For each source, similarly to [29], we assume that the exact *l* is known.

## 3 Results

### 3.1 Optimal sensing volume for a stand-alone OPM sensor

For both NMOR and SERF sensors, *L* is the length of the cell along the sensitive direction. For NMOR, *D*^2^ is the cross-section of the cell, whereas, for SERF, *D*^2^ is the cross-section of the laser beam. We impose 0.2 cm *L_opt_* 5 cm and 0.2 cm *D_opt_* 2 cm, to be compatible with the majority of cell production processes.

**Figure 2:**
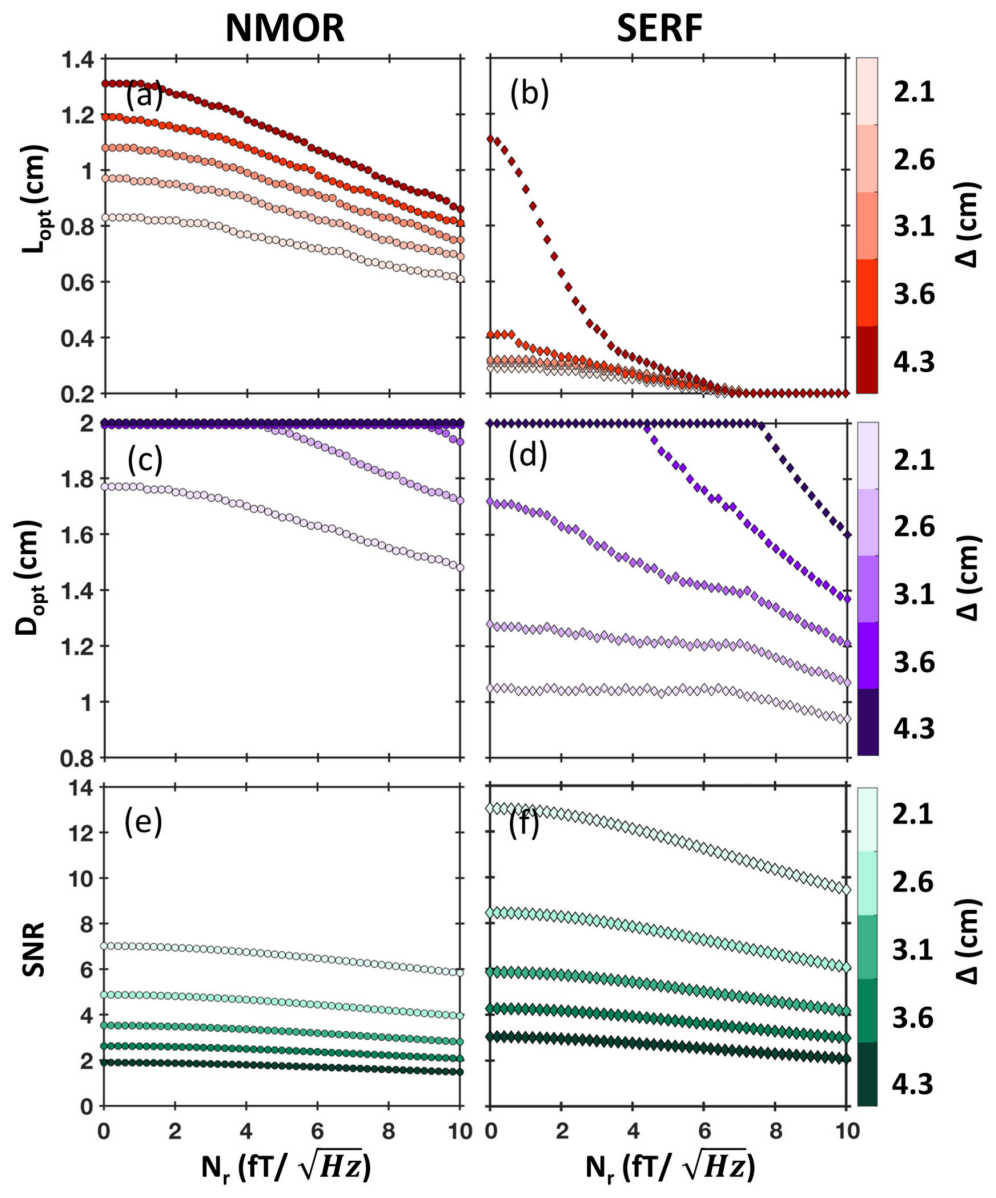
Optimal sensing element dimensions for a single OPM sensor calculated for various depths of ECD Δ, *f_BW_* =25 Hz. Optimal length *L_opt_* for corresponding *D_opt_* as a function of residual noise *N_r_* for NMOR sensor (a) and for SERF sensor (b); optimal diameter *D_opt_* for corresponding *L_opt_* as a function of *N_r_* for NMOR sensor (c) and for SERF sensor (d); SNR for corresponding *L_opt_* and *D_opt_* for NMOR sensor (e) and for SERF sensor (f).

In general, both the recorded signal *S* and the brain noise degrade with *L* because the sensor is averaging over regions increasingly far from the scalp. The intrinsic sensitivity *δB_i_* is instead lower for larger sensing volumes (Fig. A.1). *S*, *δB_i_*, ${\tilde{N}}_{b}$ decrease with different scaling with *L* and *D*. Fig. 2a–d show the optimal dimensions *L_opt_* and *D_opt_* as a function of the residual noise *N_r_* for few depths of the ECD Δ. For superficial sources (Δ < 35 mm), as *L* increases the signal decreases faster than the brain noise, and thus the optimal length varies between 0.6–1.1 cm for NMOR (Fig. 2a) and 0.2–0.3 cm for SERF (Fig. 2b). Whereas for deeper sources (Δ > 35 mm) the brain noise averages out faster than the signal of interest. Therefore, for the low-noise regime $\text{(up to }5\text{fT}\;/\sqrt{Hz}\text{ ) }$, the optimal cell length changes faster with *N_r_* for deeper sources than for the superficial ones. This effect is stronger for the SERF sensor (Fig. 2b), which has lower intrinsic noise level. In the limit of high residual noise $\left(10\text{fT}\;/\sqrt{Hz}\right)$, the optimal sensor length decreases. This is because the residual noise is independent of the cell size.

The magnetic field distribution at a given distance *a* outside of the head is relatively smooth, i.e, the magnetic field around the optimal sensor location is approximately constant (refer to topographic maps of a typical MEG data). Thus, increasing *D* improves the sensor’s sensitivity by reducing *δB_i_*. For example, an increase of D from 0.2 cm to the maximum allowed 2 cm reduces *δB_i_* by 50% for NMOR and by 90% for SERF (Appendix A), while keeping *S* and ${\tilde{N}}_{b}$ are almost constant. Overall, wider sensors (*D* > 1 cm) record higher SNR and better detect deep sources (Fig. 2c–d).

In Fig. 2e-f we show the signal-to-noise ratio SNR as a function of residual noise *N_r_* for *L_opt_* and *D_opt_* for the same depths of ECD Δ. For NMOR (SERF) sensors, the best SNR is 7 (13), obtained for superficial sources in low noise environment. This decreases to 1.5 (2) for deep sources and high *N_r_*. The lower intrinsic noise of SERF is a significant advantage in a single sensor arrangement. Note that in our simulations, the gap between the head surface and the sensing volume *a* is the same for both sensors. In practice, this gap might be few mm larger for SERF since such sensor requires hot vapour cells and thermal insulation, which will have a significant effect on SNR.

Fig. 2 serves as a guideline for choosing the optimal sensing volume dimensions depending on the experimental conditions if the number of sensors is very limited. Also, it illustrates that *L* and *D* have a clear optimal value corresponding to the balance point between how well each sensor is measuring the ECD and how well it is averaging the noise out.

### 3.2 Optimal number of OPM sensors

**Figure 3:**
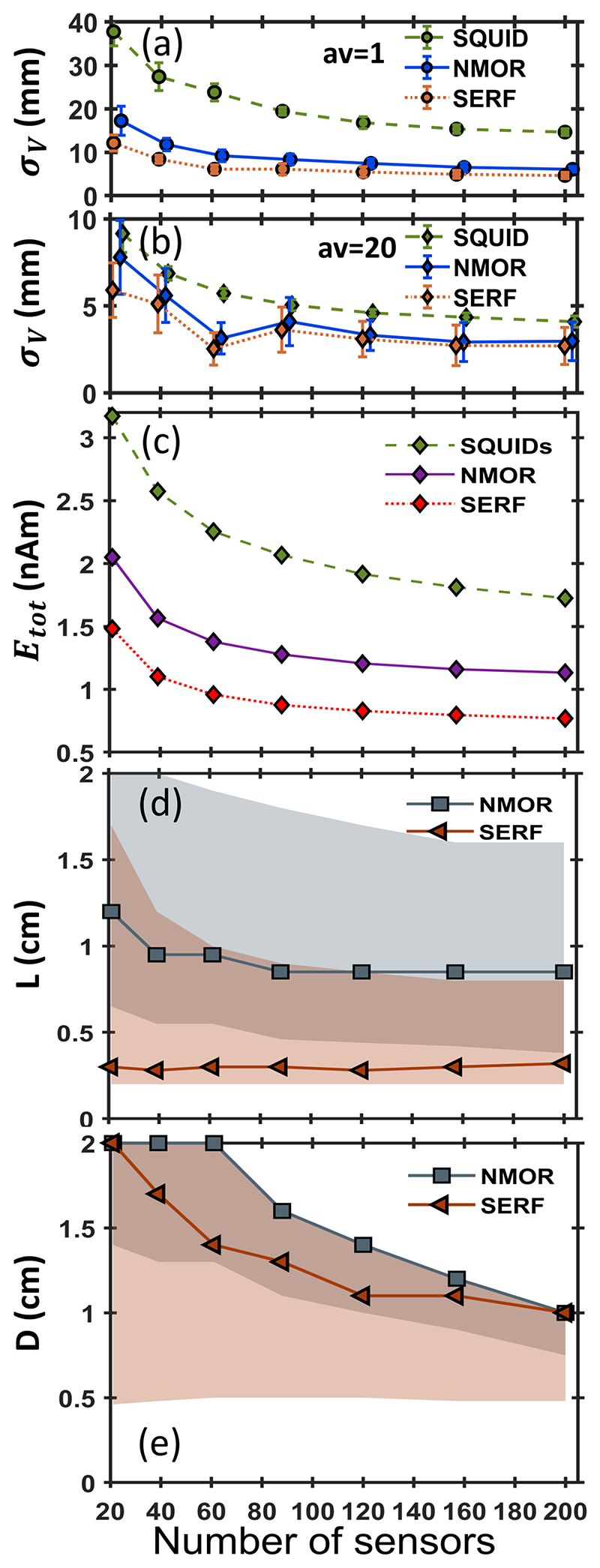
Source reconstruction accuracy of a multi-sensor array in the presence of ${\tilde{N}}_{b}$ and $N_{r}=\;5\text{fT}/\sqrt{Hz}$. (a–b) Localisation accuracy *σ_V_* as a function of the number of sensors for three exemplar arrays: Blue colour is for an NMOR OPM array with sensor dimensions set to *L* = 0.7 cm and *D* = 1.7 *cm*, orange colour is for SERF OPM array with sensor dimensions set to *L* = 0.2 cm and *D* = 1 cm. Green colour is for SQUID-MEG system with point-like magnetometers 40 mm from the head. (a) Single time-point data *av* = 1, (b) averaged 20 epochs data *av* = 20. (c) Minimum time course reconstruction accuracy *E_tot_* as a function of the number of sensors in NMOR OPM array (purple), SERF OPM array (yellow) and SQUIDs array (green). (d–e) Respectively: corresponding *L* and *D* as a function of the number of sensors. Shaded area (grey - NMOR, red - SERF) marks the range of dimensions over which *E_tot_* is within 5% of its

To evaluate the optimal number of sensors in an array, we carried out two sets of simulations. The first set, marked as *av* = 1 in Fig. 3a and Fig. 4 rows 1 and 2, is a real-time measurement where simulated data corresponds to a single epoch. The second, marked as *av* = 20 in Fig. 3b and Fig. 4 rows 3 and 4, simulates data averaged over 20 epochs. The epochs are generated for each of the 100 noise and sensors location error sets (see Section 2.2.1). As we show later, averaging over 20 epochs reduces the noise to the level, delivering almost perfect source localisation with σ_V_ 4 mm. In Fig. 3a–b, we show the localisation accuracy of a dipole placed at Δ=2.1 cm in the presence of residual noise $N_{r}=5\;\text{fT}/\sqrt{Hz}$ calculated for *L_opt_* = 0.7 cm, *D_opt_* = 1.7 cm for NMOR sensors, and *L_opt_* = 0.2 cm, *D_opt_* = 1 cm for SERF sensors. For comparison, we are also showing the localisation accuracy obtained for arrays of SQUID magnetometers. Calculations are performed for both sets, *av* = 1 (Fig. 3a) and *av* = 20 (Fig. 3b), using the procedure described in section 2.2.1. In this case, the point-like sensors are placed 4 cm from the head surface [19, 39].

To evaluate the optimal number of sensors for a time-course reconstruction, we find the minimum *E_tot_* over all the possible sensors dimensions for a given number of sensors, as shown in Fig. 3c. In Fig. 3d and e, we report the corresponding *L* and *D*. The shaded coloured areas in Fig. 3d–e mark the range of dimensions where *E_tot_* is within 5% of this minimum. For NMOR the corresponding *D* overlaps with the largest possible width for the considered number of sensors in an array. From our results it emerges that for an on-scalp OPM-MEG system with optimized sensor dimensions, the optimal number of sensors in an array is about 70. This is because this is the minimum number to reach a plateau in both *σ_V_* and *E_tot_*, indicating that no significant gain in localisation is achieved by adding more sensors. This number is slightly lower than the 100 sensors required to meet the same criteria for a conventional off-scalp MEG. [39].

#### 3.2.1 Optimal vapour cell dimensions in an array

In Fig. 4 we show how the source reconstruction accuracy depends on the sensing volume dimensions in the presence of brain noise for various powers of the residual noise. We perform the calculations for an array of 69 sensors. We choose this number because it is the closest to 70 (optimal number derived in Section 3.2) that allows the algorithm we use to equidistantly space the sensors and cover the whole upper hemisphere [50]. In Fig 4, column A represents a noise-free sensor in perfect environment ${\tilde{N}}_{r}=\;0$ fT. Column B shows low-noise regime ${\tilde{N}}_{r}=25$ fT, which represents e.g. $N_{r}=5fT\;/\sqrt{Hz}$ at measurement bandwidth of *f_BW_* = 25 Hz, conditions we chosen to investigate optimal number of sensors discussed in the previous section. Columns C and D with ${\tilde{N}}_{r}=50$ fT and ${\tilde{N}}_{r}=100$ fT respectively represent high residual noise regime.

Fig. 4 rows 1 and 2 show how *σ_V_* depends on the sensing volume dimensions in the case of real-time experiments, *av*=1. In the low residual noise regime (column B), the optimal dimensions are 0.2 cm *L_opt_* 2 cm, 0.7 cm *D_opt_* 1.6 cm for NMOR and 0.2 cm *L_opt_* 4 cm, 0.5 cm *D_opt_* 1.6 cm for SERF. With increasing amplitude of the residual noise (columns C and D), the optimal length decreases while the optimal diameter of the sensor increases. The optimal dimensions in this regime are 0.3 cm *L_opt_* 1 cm, *D_opt_* 1.2 cm for NMOR and 0.2 cm *L_opt_* 0.6 cm, *D_opt_* 0.5 cm for SERF. The white area indicates regions where the source reconstruction algorithm could not converge and the ECD could not be localised. This happens when SNR1.

In the *av*=20 set (Fig. 4 rows 3 and 4), *D* has almost no effect on *σ_V_* in the residual noise limit of 50 fT (columns A–C) for both types of sensors. NMOR arrays with *D* > 0.8 cm and *L* < 4 cm, as well as all investigated SERF arrays, reach the best localisation accuracy (*σ_V_* 4 mm). The lower limit on the volumetric error is defined by the uncertainty in the sensors location. The geometrical errors are not affected by the dimensions of the sensor. In the high residual noise regime (column D), this limit is achieved only by SERF sensors with *L* 1 cm and a narrow range of NMOR sensors (*D* > 1 cm and *L* < 0.5 cm). In all cases, *σ_V_* is better for SERF since it has lower intrinsic noise level. Considering however sensors with optimal dimensions, the difference in *σ_V_* between SERF and NMOR is significantly reduced. In Fig. 4 rows 5 and 6 we show how *E_tot_* depends on the sensing volume dimensions. In low noise limit For NMOR arrays we observe a minimum for sensors with *L* ≈ 1 cm and *D* 0.6 cm. For SERF arrays, the minimum is obtained for L1.3 cm and D0.4 cm. For higher residual noise with a standard deviation of 50 fT, *E_tot_* is stronger for *D* > 1.2 cm and 0.5 cm *L* < 1 cm in the case of NMOR and for *D* > 0.5 cm and *L* < 0.5 cm in case of SERF. When the residual noise becomes higher than the intrinsic noise level of the sensor, the dependency of *σ_V_* and *E_tot_* on the sensor diameter is lifted and the best performance is obtained with the shortest cells.

Our results show that OPM sensors achieve the best performance when the width of their sensing volume is the maximum allowed by the constraint of filling the whole head surface. For both sensor types, smallest sensors have better localisation accuracy, but relatively weak time-course reconstruction. In summary, NMOR and SERF arrays perform best when their sensing elements have *L_opt_* ≈ 0.2–1 cm and *D_opt_* 1 cm for NMOR *D_opt_* 0.6 cm for SERF. This stands for real-time and averaged data.

## 4 Discussion

In this work, we presented a model to optimise the dimensions of the sensing volume of an OPM sensor for MEG. This optimisation yields the sensing volume that delivers the best performance in a realistic scenario where both residual noise and background brain activity are present. Our model can be used as a toolkit for optimizing the design of optically pumped magnetometers in given experimental conditions. Our results show that the dimensions of the sensing element are a significant parameter to take into account while designing single OPMs or whole-head OPM-MEG systems.

Our simulations demonstrate that the optimal size of a single sensor is similar to the optimal size of a sensor in an array. This can be understood considering that the magnetic field pattern produced by a ECD outside the head features two extrema, whose magnitude and spatial distribution depend on the ECD position and orientation. Usually, only a few sensors in the array are covering the area of these maxima. Thus, the sensors required to form an optimal array have dimensions roughly similar to those of a stand-alone sensor. However, even if arrays with very few sensors can record the signal of interest with good SNR, they will not perform well in source reconstruction experiments (as one can extrapolate from Fig 3 a–b). To effectively sample the brain signal and avoid aliasing of noise coming from non-compensated sources, larger arrays are needed. Our simulations show that the optimal number of sensors in an array is around 70. Such an array reaches the best reconstruction accuracy and can directly quantify the topography of the magnetic fields produced in the brain as well as perform analysis of the recorded signals at the sensor level.

It is worth noticing that most of commercial OPM sensors have cubic cells with a side of 0.2-0.3 cm and operate in SERF regime. The intrinsic noise level of commercial sensors is calculated to be around a few $\text{fT}/\sqrt{Hz}$. The actual sensitivities $\left(\approx 10\;\text{fT}/\sqrt{Hz}\right)$ are usually not limited by fundamental noise sources, but rather by technical noise sources [38]. Columns C and D are the closest to represent such conditions in the measurement bandwidth of *f_BW_* = 25 Hz or *f_BW_* = 100 Hz respectively. According to our model, such sensors work well for reconstructing the location of the ECD in an offline experiment, where there is a number of trials to average out the noise, but can improve the performance for the time-course of the ECD or in real-time experiments. In the low noise limit the change from 0.2 cm to 1.6 cm in the width of the cell results in a 2-fold increase in the ability to extract the time course and the same increase in localization accuracy for single-trial experiments. Our simulations show that the dimensions of the sensor’s sensitive element are an important parameter to consider. Such optimisation has the potential to further improve the gain OPM-MEG systems have over the conventional SQUID-MEG. Increasing the width of the cell in the range of diameters investigated in this manuscript has no significant effect on spatial frequency sampling or spatial resolution. ^3^ Therefore, the main downside of large sensor arrays can be bulkier and heavier helmets are less ideal for wearable MEG systems.

Overall, SERF sensors deliver better performance because they have lower intrinsic noise level than NMOR sensors in similar conditions. However NMOR sensors can operate in higher magnetic field environment, have higher dynamic range and are more resilient to external field fluctuations. Furthermore, the vapour cell of SERF sensor needs to be heated to > 120°, while NMOR sensors work at room-temperature so the cell can be brought even closer to the scalp.

Note, that residual noise ${\tilde{N}}_{r}$ 50 fT eliminates both the advantage of SERF over NMOR and the advantage of large sensing volumes over small sensing volume in terms of the intrinsic noise level of the sensor. This effect is illustrated in Fig. 4 columns C and D. Furthermore, due to averaging the noises out both cell dimensions and the type of OPMs have little influence on localization accuracy (rows 3 and 4).

Our results highlight the role of brain noise, which dominates the residual noise present in a typical magnetically shielded room. Furthermore, we observed that a minimum of about 70 sensors are needed to reach best localisation accuracy independent of the sensor’s cell size and sensor type. This result is in line with the earlier hypothesis claiming that the sensor spacing should be comparable to noise correlation distance [39]. Note that without the correlated brain noise included in the model the localisation accuracy is constantly improving with an increasing number of sensors [27, 32, 39].

Our model could be expanded to optimise any other parameter that was fixed in our numerical simulations, such as the intensity of the laser beam, the atomic density or the gap between the sensor and the head. In future work, it is also desirable to refine the signal and brain noise models. For example, one could replace the spherical head model with a more realistic brain-shaped model derived from MRI scan. It would be also interesting to investigate arrays of tri-axial sensors that offer better intrinsic cancellation of the external noise sources [29]. However, in this case, a more sophisticated model for external noise is required. Ultimately, an actual recorded noise could be used to refine the optimal sensing volume dimensions.

## Supplementary Material

Appendix

## Footnotes

1 Mind the notation: the sensitivity and the amplitude spectral density have units of $\text{fT}/\sqrt{Hz}$ and is marked as *δB_x_*. The noise amplitude, i.e. the standard deviation of the noise, in a given measurement bandwidth has units of fT and is marked with $\delta {\tilde{B}}_{x}=\delta B_{x}\cdot \sqrt{f_{BW}}$. The same notation applies to *N_x_* and ${\tilde{N}}_{x}$

2 We are using the dipole fitting function ft-dipolefitting in FieldTrip, that assumes a spherical head model[45]

3 We have computed the lead field matrix from 1000 independent dipoles for an array of 69 sensors. For each sensor diameter we have computed a singular value decomposition of the lead field matrix and looked how the total spatial variability of a lead field is explained by such an array. The difference in the curves calculated for different sensor diameters were negligible in the considered range (2mm to 16 mm).
