# Supplementary material for: Optimising the sensing volume of OPM sensors for MEG source reconstruction
Source: Neuroimage. Author manuscript; Available in PMC 2023 Sep 8. (PMC7615061; doi:10.1016/j.neuroimage.2022.119747)
Supplement: Appendix [file EMS185165-supplement-Appendix.pdf]

A Appendix

A.1 NMOR OPM sensor

We optimize the sensing volume dimensions of an NMOR sensor similar to [12], with an anti-relaxation coated vapour cell containing ^{87}Rb atoms. Without a buffer gas, the atoms can travel freely in the cell and the sensing volume is $V = L \times D^2$ where L is the cell length, D^2 is the cross-section of the cell (Fig. 1). For such a sensor, the atomic shot-noise limit originates from fluctuations of the number of atoms that contribute to the signal and it depends on the finite lifetime of the light-induced atomic polarization [51],

$$\delta B_{at} = \frac{1}{\gamma}\sqrt{\frac{\Gamma}{nV}} \ , \tag{A.1.12}$$

where γ is the gyromagnetic ratio, Γ the half-width at half maximum of the NMOR resonance, and n the atomic density.

In NMOR magnetometers, the photon-shot noise is the leading contribution to the intrinsic noise. The polarization rotation noise per unit bandwidth due to quantum fluctuations is proportional to the number of photons in the probe beam N_{ph} as [51]

$$\delta B_{ph} = \frac{1}{2\gamma}\frac{\Gamma}{A_{NMOR}}\sqrt{\frac{1}{N_{ph}}} \ , \tag{A.1.13}$$

where A_{NMOR} is the amplitude of NMOR resonance.

Both δB_{at} and δB_{ph} depend on the width of the resonance Γ, which is the inverse of the spin relaxation time [52]. The width of the resonance is determined by the sum of several relaxation rates:

$$\Gamma = R_{se} + R_{wall} + R_{light} + R_{noise} \tag{A.1.14}$$

where R_{se} is the spin-exchange collision relaxation rate, R_{wall} is the wall collision relaxation rate, R_{light} is the light-induced relaxation rate, and R_{noise} is the relaxation rate induced by external field fluctuation. The relaxation rate due to collisions between alkali atoms is given by

$$R_{se} = q(I)\sigma_{se} v_{rel} n \ , \tag{A.1.15}$$

where $q(I) = (6I + 1)/(\pi \times (8I + 4)))$ is the nuclear slow-down factor, I the nuclear spin of alkali atom, σ_{se} is the spin-exchange collision cross-section and $v_{rel} = \sqrt{2} v_{th}$ is the average atomic relative velocity with $v_{th} = \sqrt{8k_B T/m}$, where k_B is the Boltzmann constant, T the temperature of the gas and m the atomic mass.

The wall-collision-induced relaxation rate is given by:

$$R_{wall} = C_w \frac{1}{T_c} \ , \tag{A.1.16}$$

where C_w is the probability of electron spin relaxation during the collisions with the coating of the walls and $T_c = 4V/(v_{th} S_{cell})$ with S_{cell} as the surface area of the cell.

The light-induced relaxation rate is caused by the pump light [3] and is due to transitions between Zeeman ground states. This rate is proportional to the average incident light intensity I_{mean}:

$$R_{light} = 2 \frac{k_\nu}{\nu_l} I_{mean} \tag{A.1.17}$$

where k_ν is the microscopic absorption cross-section, the reduced Planck constant and ν_l the frequency of the pumping light.

Finally, spatial field fluctuations randomly shift the resonance frequency, resulting in a broadening of the magnetic resonance lines according to

$$R_{noise} = \gamma^2 \left(\Delta B\right)^2 \frac{L}{v_{th}} \tag{A.1.18}$$

where ΔB is the root mean square of the magnetic field fluctuations over the length of the cell L. For a sensor similar to [12], ΔB is dominated by the bias field applied over the cell and is thus independent of the spatial fluctuations of measured signals.

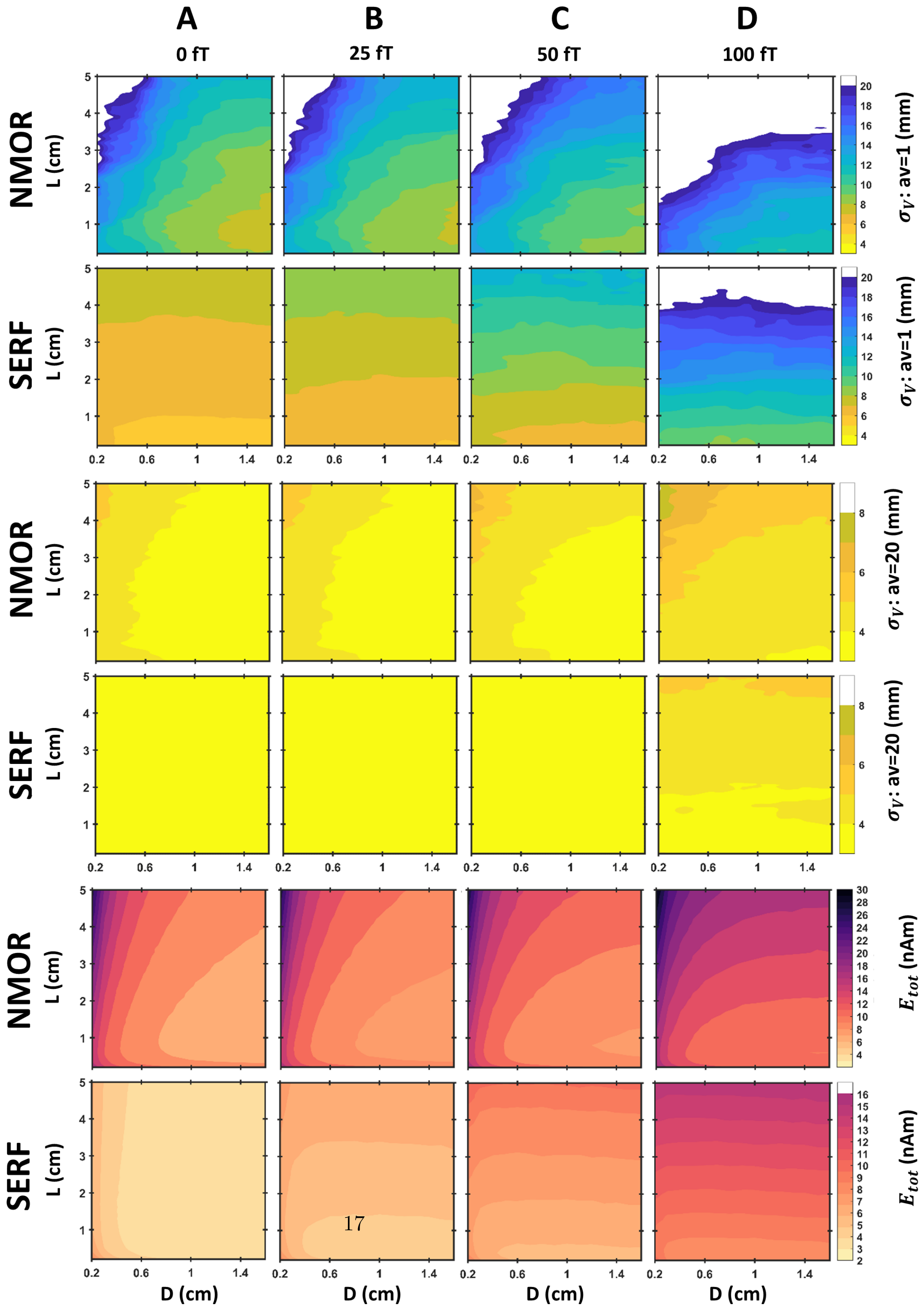

Figure 4: Source reconstruction accuracy as a function of L and D in the presence of brain noise $\tilde{N}_b$ with residual noise standard deviation set to $\tilde{N}_r$ =0 fT (column A), $\tilde{N}_r$ =25 fT (column B), $\tilde{N}_r$ =50 fT (column C) and $\tilde{N}_r$ =100 fT [illegible]

Table A.1: Constants and parameters used in the model (1) [53], (2) [52], (3) [54], (4) [3], and (5) [55].

Common			
k_ν	$1.12 \cdot 10^{-23}$ m^2 (1)		
ν_{laser}	377 THz		
P_{laser}	$7 \cdot 10^{-6}$ W		
f_{BW}	25 Hz		
ΔB	1 nT/m		
NMOR OPM (^{87}Rb)		SERF OPM (^{87}Rb)	
γ	$43 \cdot 10^9$ Hz/T	γ^e	$1.7 \cdot 10^{11}$ Hz/T (5)
n	$8.5 \cdot 10^{15}$ m^{-3}	n	$1 \cdot 10^{20}$ m^{-3}
I	3/2	I	3/2
T	295 K	T	450 K
A_{NMOR}	$0.6 \cdot 10^{-3}$ rad	σ_{SD}^{SERF}	$1.6 \cdot 10^{-21}$ m^{-2} (5)
σ_{se}	$2 \cdot 10^{-18}$ m^2 (3)	σ_{sd}^{Ne}	$1 \cdot 10^{-28}$ m^{-2} (5)
C_w	10^{-4} (4)	$\sigma_{sd}^{N_2}$	$1 \cdot 10^{-26}$ m^{-2} (5)
		D_0	$0.2 \cdot 10^{-4}$ m^2/s (5)
		p_{Ne}	600 Torr
		p_{N_2}	20 Torr

A.2 SERF OPM sensor

We model a SERF OPM with a single-beam configuration similar to commercial sensors [56]. The vapour cell contains ^{87}Rb atoms, Ne as a buffer gas, and N_2 as quenching gas. For the optimal pumping rate, the atomic shot noise is given by [3, 56]

$$\delta B_{at} = \frac{4}{\tilde{\gamma^e}}\sqrt{\frac{\Gamma}{nV}} \tag{A.2.1}$$

where $\tilde{\gamma^e} = \frac{\gamma^e}{2I+1}$ and γ^e is the gyromagnetic ratio of a bare electron. V is the volume corresponding to the intersection of a laser beam with the vapour cell volume $V = L \cdot D^2$, where L is the cell length, D^2 is the laser beam cross-section area; and the photon shot-noise is

$$\delta B_{ph} = \frac{4\Gamma}{\tilde{\gamma^e}}\sqrt{\frac{1}{N_{ph}}} \ . \tag{A.2.2}$$

In the SERF magnetometer, spin-exchange relaxation is suppressed. The residual magnetic width Γ is due to spin-destruction collisions R_{SD}, collisions with the walls R_{wall}, the interaction with the light R_{pump}. With optimal pumping R_{pump} equals the sum of R_* caused by all other relaxation mechanisms. Overall the relaxation rate in SERF regime is expressed as:

$$\Gamma = R_{SD} + R_{wall} + R_{pump} + R_{noise} \ , \tag{A.2.3}$$

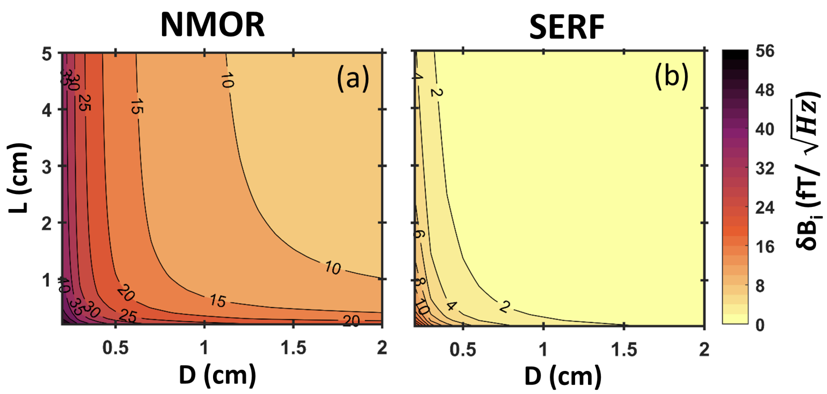

Figure A.1: OPM intrinsic sensor sensitivity δB_i as a function of L and D of the sensing volume for NMOR (a) and-https://www.overleaf.com/project/6037b22e4e706d24aa788acc for SERF (b)

where the R_{SD} is the relaxation rate due to spin destruction is given by

$$R_{SD} = R_{SD}^{SERF} + R_{SD}^{B} + R_{SD}^{Q} \ . \tag{A.2.4}$$

Here the first term is due to collisions with other alkali atoms, the second term is due to collisions with buffer gas atoms, and the third term is due to collisions with quenching gas molecules. Each of these three terms can be written as

$$R_{SD}^{*} = \frac{1}{2I+1} n_* v_{rel} \sigma_{SD}^{*} \ , \tag{A.2.5}$$

where ^{o} denotes relevant atoms, $v_{rel} = \sqrt{8k_BT/(\pi M)}$, $M = (1/m + 1/m')^{-1}$ with m alkali atom mass, m' the relevant atom mass, n_* is relevant atomic density.

The wall-collision-induced relaxation rate is given by

$$R_{wall} = D_0 \frac{p_0}{p} \left(\frac{2.4^2}{D^2} + \frac{\pi^2}{L^2} \right) \ , \tag{A.2.6}$$

where D_0 is the diffusion constant of the alkali atom within the buffer gas, $p_0 = 760$ Torr, p is the buffer gas pressure.

The broadening due to magnetic field fluctuations are the same for SERF and NMOR, thus R_{noise} is obtained from Eq. A.1.18.
